# Transgenerational Transmission of Enhanced Ocular Dominance Plasticity from Enriched Mice to Their Non-enriched Offspring

**DOI:** 10.1523/ENEURO.0252-18.2018

**Published:** 2019-02-05

**Authors:** Evgenia Kalogeraki, Rashad Yusifov, Siegrid Löwel

**Affiliations:** 1 Department of Systems Neuroscience, J.F.B. Institut für Zoologie und Anthropologie, 37075 Göttingen; 2 Göttingen Graduate School of Neurosciences, Biophysics and Molecular Biosciences, Universität Göttingen, 37073 Göttingen, Germany; 3 Campus Institute for Dynamics of Biological Networks, 37075 Göttingen, Germany

**Keywords:** enriched environment, mouse, ocular dominance plasticity, optical imaging, transgenerational, visual cortex

## Abstract

In recent years, evidence has accumulated that non-Mendelian transgenerational inheritance of qualities acquired through experience is possible. In particular, it has been shown that raising rodents in a so-called enriched environment (EE) can not only modify the animals’ behavior and increase their susceptibility to activity-dependent neuronal network changes, but also influences both behavior and neuronal plasticity of the non-enriched offspring. Here, we tested whether such a transgenerational transmission can also be observed in the primary visual cortex (V1) using ocular dominance (OD) plasticity after monocular deprivation (MD) as a paradigm. Whereas OD plasticity after 7 d of MD is absent in standard-cage (SC) raised mice beyond postnatal day (P)110, it is present lifelong in EE-raised mice. Using intrinsic signal optical imaging to visualize cortical activity, we confirm these previous observations and additionally show that OD plasticity is not only preserved in adult EE mice but also in their adult non-enriched offspring: mice born to enriched parents, but raised in SCs at least until P110 displayed similar OD shifts toward the open eye after 7 d of MD as age-matched EE-raised animals. Furthermore, testing the offspring of EE-female versus EE-males with SC-mating partners revealed that only pups of EE-females, but not of EE-males, preserved OD plasticity into adulthood, suggesting that the life experiences of the mother have a greater impact on the continued V1 plasticity of the offspring. The OD plasticity of the non-enriched pups of EE-mothers was, however, mechanistically different from that of non-enriched pups of EE-parents or EE mice.

## Significance Statement

Recently evidence is accumulating that life experiences and thus acquired qualities of parents can be transmitted across generations in a non-Mendelian fashion and have a significant impact on the fitness of offspring. Raising mice in a so-called enriched environment with enhanced opportunities for social interaction, voluntary physical exercise, and explorative behavior has been shown to boost cortical plasticity. Our results now show that the plasticity-promoting effect of enrichment on ocular dominance plasticity, a well-established plasticity paradigm in a primary sensory cortex, can also be transmitted from enriched parents to their non-enriched offspring. Thus cortical plasticity is not only influenced by an animal’s life experiences but can also be modified by the life experiences of its parents.

## Introduction

During brain development, experience is continuously interacting with genetic information to shape and functionally optimize neuronal circuits. The environmental conditions under which animals grow up therefore exert a powerful influence on the functioning of their brain and behavior. There is a rich and growing body of evidence documenting changes across molecular, anatomical, and functional levels when animals raised in a so-called enriched environment (EE) are compared with animals housed in standard cages (SCs; Sale et al., 2014). Enriched conditions refer to paradigms in which animals are housed in larger groups in bigger and spatially complex cages equipped with mazes, toys, and running wheels. In this environment, animals experience a “combination of complex inanimate and social stimulation” (Rosenzweig et al., 1962) because of enhanced opportunities to engage in voluntary physical, social, and cognitive stimulation (van Praag et al., 2000; Löwel et al., 2017). In rodents, enrichment alters the expression of key signaling molecules involved in regulating brain excitability and plasticity (Cancedda et al., 2004): BDNF and serotonin levels are increased (Cancedda et al., 2004; Sale et al., 2004, 2007; Baroncelli et al., 2010), GABAergic inhibition is reduced (Greifzu et al., 2014) and the density of extracellular matrix perineuronal nets is reduced (Sale et al., 2007). Furthermore, EE prolongs and restores ocular dominance (OD) plasticity in the visual cortex into old age (Baroncelli et al., 2010; Greifzu et al., 2014, 2016), increases the volume of many brain areas (Diamond et al., 1964), and notably also alters maternal behavior (Sale et al., 2004).

In addition to these “immediate” effects on brain functioning and plasticity, exposure to EE can also influence brain plasticity and behavior of the next generation (Arai and Feig, 2011). Early studies had already documented that exposure of pregnant rats to an EE enhanced not only their ability to find their way in a maze, but also the performance of their offspring in the same task (Kiyono et al., 1985). Furthermore, juvenile enrichment [EE from postnatal day (P)14–P28] not only rescued a genetic defect in long-term potentiation and memory formation in the short-term enriched mice, but also in their 4-week-old non-enriched offspring (Arai et al., 2009). EE during the first days of life was shown to accelerate visual system development because pups of EE-mice receive higher levels of maternal care, continuous physical contact, and more licking compared with SC-reared pups (Cancedda et al., 2004). Interestingly, the licking-grooming behavior itself is heritable: the offspring of high and low frequent licking-grooming mothers become high and low frequent licking-grooming mothers, respectively (Arai and Feig, 2011).

Motivated by these studies, we analyzed whether there is also transgenerational transmission of increased plasticity in a primary sensory cortex and chose the mouse primary visual cortex (V1) as our model system. OD plasticity in V1 induced by monocular deprivation (MD) is a well established model system to study cortical plasticity (Wiesel and Hubel, 1963). Briefly occluding one eye causes a shift in the OD toward the open eye (Wiesel and Hubel, 1963; Dräger, 1978). In SC-raised mice, OD plasticity is most pronounced in juvenile animals during the critical period (P25–P35), reduced in young adult animals, and not detectable in animals beyond P110 after 7 d of MD (Lehmann and Löwel, 2008; Espinosa and Stryker, 2012). In contrast, mice raised in EE-cages or transferred to EE after P110 display lifelong OD plasticity (Greifzu et al., 2014, 2016).

Here we tested whether the plasticity-promoting effect of EE on OD plasticity is also present in non-enriched SC-raised pups of EE mice. To this end, pregnant EE-mothers were transferred to SCs, the offspring were raised in SCs into adulthood (>P110), and then OD plasticity was analyzed after 7 d of MD using intrinsic signal optical imaging (Cang et al., 2005). During MD, spatial vision of the open eye was quantified daily by optometry (Prusky et al., 2004, 2006). OD plasticity was preserved in the offspring of EE-parents and EE-mothers, but not in offspring of EE-fathers. Whereas the OD shift of non-enriched offspring of EE-parents was mediated by a reduction of V1 activation through the deprived eye, as typically seen in adult EE-mice (Greifzu et al., 2014; and present study), OD shifts of the offspring of EE-mothers were mechanistically different. Our data clearly show that even in a primary sensory cortex, the plasticity promoting effect of growing up in a stimulating (or less deprived) environment not only affects the mice experiencing this enrichment, but also their non-enriched offspring, and enriched mothers seem to have a bigger impact on the offspring’s cortical plasticity.

## Materials and Methods

### Animals and rearing conditions

C57BL/6 mice were obtained from the mouse colony of the central animal facility of our university, and housed with a 12 h light/dark cycle, with food and water available *ad libitum*. All experimental procedures comply with National Institutes of Health guidelines for the use of animals.

Mice were housed in either normal SC or EE cages. EE cages [Marlau, Viewpoint; 56 × 37 × 32 cm (L × W × H)] are approximately nine times larger than our SCs [26 × 20 × 14 cm [L × W ×H )], with two floors, providing mice a bigger exploration area, more opportunities for social interaction (16 mice compared with 3–4 in SCs) and physical exercise (3 running wheels). The lower compartment is divided in two areas: the “living area” with three running wheels for physical exercise and a red tunnel to protect the animals from light during the day, and the “food area” where food is located. To move from the “living area” to the “food area”, mice have to go to the upper compartment using the ladder, pass through the maze, and slide down through a tube. They can return to the “living area” through a revolving door which opens only in one direction, thus they are forced to move through the maze again to get food. The maze was changed three times per week, and there were in total 12 different configurations. For the initial comparison of OD plasticity in adult (>P110) mice, we used either EE- or SC-raised mice. Mice in these groups were born and raised in EE or SC cages until the experiment. For testing whether increased plasticity can be transferred to the next generation, mating of EE-mice took place in EE-cages and pregnant females were then transferred to SCs 5–7 d before delivery.

Furthermore, we crossed EE-males with SC-females as well as SC-males with EE-females. The mating always happened in the cage of the female mouse. Pregnant EE-mothers were transferred to SCs (or stayed in SCs for the SC-mother/EE-father pairing), the offspring were raised in SCs into adulthood (>P110), and then OD plasticity was analyzed after 7 d of MD using intrinsic signal optical imaging (Cang et al., 2005).

In summary, the following five experimental groups were analyzed for the present study: (1) SC-raised mice (SC; *n* = 8, age range P137–P200), (2) EE-raised mice (EE; *n* = 8, age range P161–P168), (3) SC-raised mice from parents raised in EE (EE-parents; *n* = 10, age range P118–P261), (4) SC-raised mice from fathers raised in EE and mothers raised in SC (EE-father; *n* = 10, age range P127–P194), and (5) SC-raised mice from mothers raised in EE and fathers raised in SC (EE-mother; *n* = 11, age range P142–P188; Table 1). Mice within each experimental group were from 2 to 3 litters with different parents and were assigned to MD and noMD conditions to ensure littermate controls.

**Table 1:** Ages and ODIs of experimental animals

**Figure**	**Group**	**Mouse**	**Age, d**	**ODI**
1	SC/noMD	1	137	0.34
		2	139	0.33
		3	151	0.27
		4	200	0.31
	SC/MD	1	152	0.19
		2	154	0.30
		3	155	0.23
		4	195	0.37
	EE/noMD	1	164	0.32
		2	161	0.26
		3	161	0.20
		4	168	0.20
	EE/MD	1	163	−0.01
		2	164	0.12
		3	164	0.07
		4	164	−0.03
2	EE-parents/noMD	1	119	0.32
		2	125	0.24
		3	209	0.17
		4	205	0.23
	EE-parents/MD	1	118	0.11
		2	126	0.10
		3	130	0.16
		4	261	0.07
		5	200	0.05
		6	208	0.13
3	EE-fathers/noMD	1	127	0.16
		2	135	0.24
		3	136	0.21
		4	188	0.25
		5	184	0.32
	EE-fathers/MD	1	132	0.19
		2	132	0.12
		3	133	0.20
		4	136	0.20
		5	194	0.23
	EE-mothers/noMD	1	142	0.26
		2	143	0.33
		3	168	0.28
		4	169	0.22
		5	151	0.21
		6	188	0.27
	EE-mothers/MD	1	166	0.16
		2	174	0.15
		3	175	0.11
		4	176	0.04
		5	177	0.15

Age and ODI of all experimental animals of each experimental group, subdivided by figure in which data were displayed.

### Monocular deprivation

The animals’ right eye was deprived for 7 d according to published protocols (Gordon and Stryker, 1996). Briefly, mice were anesthetized using 2% isoflurane in a mixture of O_2_ and N_2_O (1:1), the eyelids were trimmed and sutured together. Mice were returned to their home cages for recovery and checked daily to ensure that the eyes remained closed for the following 7 d.

### Virtual reality optomotor test

To check visual capabilities and the effectiveness of the MD, both the spatial frequency and the contrast sensitivity threshold of the optomotor reflex of all mice was measured daily using the virtual reality optomotor system (Prusky et al., 2004). Briefly, freely moving mice were positioned on a small elevated platform surrounded by four computer monitors (33.5 × 26.5 cm) forming a box. A rotating virtual cylinder, composed of a vertical sine wave grating, was projected on the screens. Parameters including spatial frequency, contrast and speed of the moving sine wave grating could be varied by the experimenter. On detecting the stimulus, the mouse will reflexively track the grating by moving the head in the direction of rotation. Spatial frequency thresholds at full contrast and contrast thresholds at six different spatial frequencies [0.031, 0.064, 0.092, 0.103, 0.192, 0.272 cycles/degree (cyc/deg)] were measured daily, before and after MD (7 d total).

### Optical imaging of intrinsic signals and visual stimuli

#### Surgery

Mice were anesthetized with 2% halothane in O_2_:N_2_O (1:1) and injected with rimadyl (0.1 mg/mouse, s.c.; Pfizer), atropine (0.3 mg/mouse, s.c.; Franz Köhler), dexamethasone (0.2 mg/mouse, s.c.; Ratiopharm), and chlorprothixene (0.2 mg/mouse, i.m.; Sigma-Aldrich). After placing animals in a stereotaxic frame, anesthesia was maintained with 0.8% halothane in a mixture of O_2_:N_2_O (1:1). A small incision of the skin was made to expose visual cortex and low-melting point agarose (2.5% in 0.9% NaCl) and a glass coverslip were placed over the exposed area. In case of an MD mouse, the sutures were removed to open the deprived eye.

#### Data acquisition and visual stimulation

Mouse V1 responses were recorded through the skull using the “Fourier”-imaging method (Kalatsky and Stryker, 2003), optimized for the assessment of OD plasticity (Cang et al., 2005). V1 signals were visualized with a CCD-camera (Dalsa 1M30) using a 135 × 50 mm tandem lens configuration (Nikon), with red illumination light (610 ± 10 nm). Active brain regions absorb more of the red light and appear darker in the images. Frames were acquired at a rate of 30 Hz, temporally binned to 7.5 Hz, and stored as 512 × 512 pixel images after spatial binning of the camera image.

Visual stimuli were presented on a high refresh rate monitor (Hitachi, ACCUVUE, HM-4921-D, 21 inch) positioned 25 cm from the eyes. Stimuli consisted of white drifting horizontal bars (2° wide). The amplitude component of the optical signal represents the intensity of neuronal activation (expressed as fractional change in reflectance −10^−4^) and was used to calculate OD. At least three maps per animal were averaged to compute the OD index (ODI) as (C − I)/(C + I), with C and I representing the response magnitudes of each pixel to visual stimulation of the contralateral and ipsilateral eye. The ODI ranges from −1 to +1, with negative values representing ipsilateral and positive values representing contralateral dominance. Note that the V1 activity maps illustrated in the results section are the averages of at least three maps from individual animals. OD maps were always assessed and quantified by an experimenter blind to the animals’ or its parents’ rearing conditions.

### Statistical analysis

All intragroup and intergroup comparisons were analyzed by ANOVA followed by Bonferroni *post hoc* test. The intergroup comparison of the enhancement of the spatial frequency and contrast sensitivity thresholds were analyzed by two-way ANOVA with repeated measurements and Bonferroni correction. Normal distribution of data was checked using Shapiro–Wilk test. Data were analyzed using GraphPad Prism 7 and IBM SPSS statistics. The levels of significance were set as *p < 0.05, **p < 0.01, ***p < 0.001. Data are represented as means ± SEM (Table 2).

**Table 2:** Statistical analysis

**Fig.**	**Group**	**Parameter**	***N***	**CI_95_**	**Data structure**	**Comparison**	**Type of test**	***p* value**
1*B*	SC/EE	[A] SC-no MD[B] SC-MD[C] EE-no MD[D] EE-MD	4444	(0.2632;0.3618)(0.1463;0.3987)(0.1536;0.3364)(−0.0738;0.1488)	Normal distributionNormal distributionNormal distributionNormal distribution	A vs BC vs DB vs D	Two-way ANOVA with Bonferroni’s multiple comparisons	0.38370.0037**0.001**
1*C*	SC/EE	[A] SC-no MD-Contra(C)[B] SC-no MD-Ipsi (I)[C] SC-MD-Contra (C)[D] SC-MD-Ipsi (I)[E] EE-no MD-Contra(C)[F ] EE-no MD-Ipsi (I)[G] EE-MD-Contra (C)[H] EE-MD-Ipsi (I)	44444444	(1.179;1.716)(0.6298;0.9052)(1.248;2.052)(0.5893;1.526)(1.778;2.666)(1.25;1,73)(1.41;2.08)(1.219;2.156)	Normal distributionNormal distributionNormal distributionNormal distributionNormal distributionNormal distributionNormal distributionNormal distribution	A vs BA vs CB vs DC vs DE vs FE vs GF vs HG vs H	Mixed ANOVA with Bonferroni’s multiple comparisons	0.0064**0.90710.62890.0228*0.003**0.0343*0.9179>0.999
2*B*	no MD/ MD	[A] no MD[B] MD	46	(0.1491;0.3459)(0.06253;0.1451)	Normal distributionNormal distribution	A vs B	Unpaired *t* test	0.0019**
2*C*	no MD/ MD	[A] no MD-Contra (C)[B] no MD-Ipsi (I)[C] MD-Contra (C)[D] MD-Ipsi(I)	4466	(1.604;2.424)(0.8888;1.506)(1.055;1.718)(0.899;1.581)	Normal distributionNormal distributionNormal distributionNormal distribution	A vs BA vs CB vs DC vs D	Two-way ANOVA with Bonferroni’s multiple comparisons	0.0062**0.0237*0.997>0.999
3*B*	EE fathers/EE mothers	[A] EE fathers-no MD[B] EE fathers-MD[C] EE mothers-no MD[D] EE mothers- MD	5565	(0.1633;0.3087)(0.1373;0.2387)(0.216;0.3074)(0.06029;0.1837)	Normal distributionNormal distributionNormal distributionNormal distribution	A vs BC vs D	Two-way ANOVA with Bonferroni’s multiple comparisons	0.81010.0011**
3*C*	EE fathers/EE mothers	[A] EE fathers-no MD-Contra(C)[B] EE fathers-no MD-Ipsi (I)[C] EE fathers-MD-Contra (C)[D] EE fathers-MD-Ipsi (I)[E]EE mothers-no MD-Contra(C)[F ]EE mothers-no MD-Ipsi (I)[G] EE mothers-MD-Contra (C)[H] EE mothers-MD-Ipsi (I)	55556655	(1.25;2.478)(0.7768;1.655)(0.712;2.504)(0.6932;1.759)(1.519;2.101)(0.9971;1.536)(1.351;2.757)(1.202;2.266)	Normal distributionNormal distributionNormal distributionNormal distributionNormal distributionNormal distributionNormal distributionNormal distribution	A vs BA vs CB vs DC vs DE vs FE vs GF vs HG vs H	Mixed ANOVA with Bonferroni’s multiple comparisons	0.0026**0.98500.96890.0537<0.0001***0.98540.05150.9495
4*A*	EE parents	[A] no MD[B] MD	46	(0.379;0.3823)(0.3985;0.437)	Normal distributionNormal distribution	A vs B	One-way ANOVA	0.0011**
4*A*	EE fathers	[A] no MD[B] MD	55	(0.3792;0.3801)(0.4025;0.4498)	Normal distributionNormal distribution	A vs B	One-way ANOVA	0.0007***
4*A*	EE mothers	[A] no MD[B] MD	55	(0.387;0.391)(0.3985;0.4297)	Normal distributionAssume normality	A vs B	One-way ANOVA	0.0043**
4*B*	EE parents/ EE fathers/EE mothers	[A] EE parents MD[B] EE fathers MD[C] EE mothers MD	655	(4.518;14.58)(5.862;18.28)(3.593;11.68)	Normal distributionNormal distributionNormal distribution	A vs BA vs CB vs C	One-way ANOVA	>0.999>0.9990.4994
4*C*	EE parents-no MD	[A] Day 0[B] Day 7	44	(4.368;12.56)(4.406;12.68)	Normal distributionNormal distribution	A vs B at 0.031A vs B at 0.064A vs B at 0.092A vs B at 0.103A vs B at 0.192A vs B at 0.272	Two-way ANOVA with Bonferroni’s multiple comparisons	>0.999>0.999>0.999>0.999>0.999>0.999
4*D*	EE parents-MD	[A] Day 0[B] Day 7	66	(4.266;12.42)(5.582;24.46)	Normal distributionNormal distribution	A vs B at 0.031A vs B at 0.064A vs B at 0.092A vs B at 0.103A vs B at 0.192A vs B at 0.272	Two-way ANOVA with Bonferroni’s multiple comparisons	0.7252<0.0001***<0.0001***<0.0001***<0.0001***>0.999
4*E*	EE fathers-no MD	[A] Day 0[B] Day 7	55	(4.211;12.99)(4.229;13.12)	Normal distributionNormal distribution	A vs B at 0.031A vs B at 0.064A vs B at 0.092A vs B at 0.103A vs B at 0.192A vs B at 0.272	Two-way ANOVA with Bonferroni’s multiple comparisons	>0.999>0.999>0.999>0.999>0.999>0.999
4*F*	EE fathers-MD	[A] Day 0[B] Day 7	55	(4.14;12.83)(5.347;23.99)	Normal distributionNormal distribution	A vs B at 0.031A vs B at 0.064A vs B at 0.092A vs B at 0.103A vs B at 0.192A vs B at 0.272	Two-way ANOVA with Bonferroni’s multiple comparisons	0.1166<0.0001***<0.0001***<0.0001***<0.0001***0.2105
4*G*	EE mothers- no MD	[A] Day 0[B] Day 7	66	(4.061;14.5)(4.024;14,53)	Normal distributionNormal distribution	A vs B at 0.031A vs B at 0.064A vs B at 0.092A vs B at 0.103A vs B at 0.192A vs B at 0.272	Two-way ANOVA with Bonferroni’s multiple comparisons	>0.999>0.999>0.999>0.999>0.999>0.999
4*H*	EE mothers-MD	[A] Day 0[B] Day 7	55	(4.076;14.61)(5.064;21.3)	Normal distributionNormal distribution	A vs B at 0.031A vs B at 0.064A vs B at 0.092A vs B at 0.103A vs B at 0.192A vs B at 0.272	Two-way ANOVA with Bonferroni’s multiple comparisons	0.2940<0.0001***<0.0001***<0.0001***<0.0001***>0.3072

The columns in the table from left to right refer to the figures, the groups compared, parameters analyzed, number of animals (*N*), lower and upper 95% confidence interval of the mean (CI_95_), distribution of the values (data structure), comparisons of (sub)groups abbreviated as indicated in the parameter column (comparison), test applied for the comparison, and statistical readout (*p* value). Significance levels were set as *p < 0.05, **p < 0.01, ***p < 0.001.

## Results

### OD plasticity is preserved in adult EE-raised but not in adult SC-raised mice

**Figure 1:**
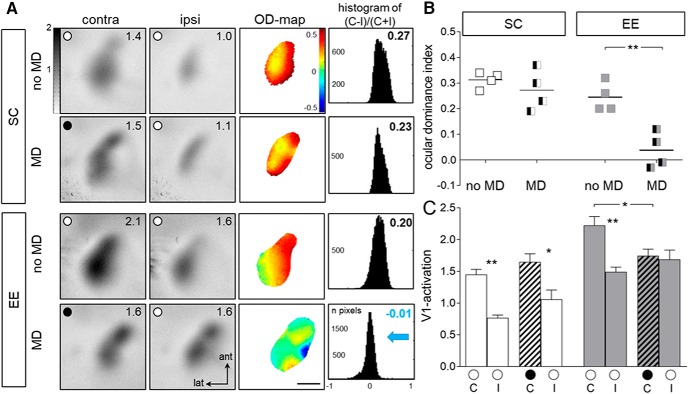
**. OD plasticity is preserved in V1 of old EE-mice. *A***, Examples of optically recorded activity maps induced by visual stimulation of the contralateral and ipsilateral eye in the binocular part of V1 of SC- and EE-raised mice, without (no MD) or after 7 d of MD. Gray scale-coded activity maps [numbers in the top right corner correspond to the quantified V1 activation (×10^−4^); see gray scale, left], color-coded two-dimensional OD maps (color codes ODI; see scale to the right of OD map), and the histogram of OD scores, including the average ODI, are illustrated. MD eye is indicated by the black circle in the V1 map, open circles indicate an open eye. In both SC- and EE-mice without MD (no MD), the activity patch evoked by visual stimulation of the contralateral eye is darker than the one of the ipsilateral eye, warm colors prevail in the two-dimensional OD maps and ODI values are positive. After MD, V1 activation changes in EE mice but not in SC mice. Whereas V1 of SC mice remained dominated by the deprived (contralateral) eye, there was an OD shift toward the open eye in V1 of EE mice: after MD, V1 of EE mice was now less strongly activated by the contralateral eye so that both eyes activated V1 similarly, colder colors appeared in the OD maps, and the ODI values were lower, i.e., the ODI histograms shifted to the left (blue arrow). ant, Anterior; lat, lateral. Scale bar, 1 mm. ***B***, ***C***, Quantification of visual cortical activation before and after MD. ODI (***B***) and V1 activation (***C***) are illustrated. ***B***, Optically imaged ODIs without (no MD) and with MD: symbols represent ODI values of individuals, means are marked by horizontal lines. MD is indicated by half-black squares. ***C***, V1 activation elicited by stimulation of the contralateral (C) or ipsilateral (I) eye. Hatched bar indicates MD eye. Data represented as mean ± SEM. Statistical significance was calculated using ANOVA and *p* values were corrected for multiple comparisons. *p < 0.05, **p < 0.01.

It was previously reported that OD plasticity is an age-dependent process (Espinosa and Stryker, 2012) because SC-raised mice no longer exhibit OD shifts after 7 d of MD when they are older than P110 (Lehmann and Löwel, 2008). In contrast, mice raised in EE display lifelong OD plasticity (Greifzu et al., 2014, 2016). Here, we confirm these previous observations by comparing OD plasticity after 7 d of MD in adult mice raised in either SCs (age range P137–P200) or EE cages (age range P161–P168; Fig. 1) using intrinsic signal optical imaging (Cang et al., 2005). In SC-mice, V1 was dominated by the contralateral eye, and remained dominated by the contralateral (deprived) eye also after MD (contralateral V1 activation noMD/MD: 1.45 ± 0.08/1.65 ± 0.13, *p* = 0.907, ANOVA; ipsilateral V1 activation noMD/MD: 0.77 ± 0.04/1.06 ± 0.15, *p* = 0.6298, ANOVA). In contrast, in EE-mice, V1 activation through the (previously) deprived eye was significantly reduced after MD (contralateral noMD/MD: 2.22 ± 0.14/1.74 ± 0.11, *p* = 0.034, ANOVA; ipsilateral noMD/MD: 1.49 ± 0.08/1.68 ± 1.22; *p* = 0.917, ANOVA; Fig. 1*A*,*C*). Accordingly, the average ODI of SC-mice without MD was 0.31 ± 0.02 (*n* = 4), and did not change after MD (ODI: 0.27 ± 0.04, *n* = 4; *p* = 0.38, ANOVA). In contrast, age-matched EE-mice showed a significant OD shift after MD: the average ODI of adult EE-mice decreased from 0.25 ± 0.03 (*n* = 4) without MD to 0.04 ± 0.03 after MD (*n* = 4; *p* = 0.0037, ANOVA; Fig. 1*B*), and average ODI values after MD were significantly different between SC- and EE-mice (*p* = 0.001, ANOVA). Notably, the OD shift of EE-mice was mediated by a reduction of V1 activation after visual stimulation of the contralateral eye, as shown in previous publications on EE-mice (Greifzu et al., 2014).

### OD plasticity was present in non-enriched offspring of EE-parents

**Figure 2:**
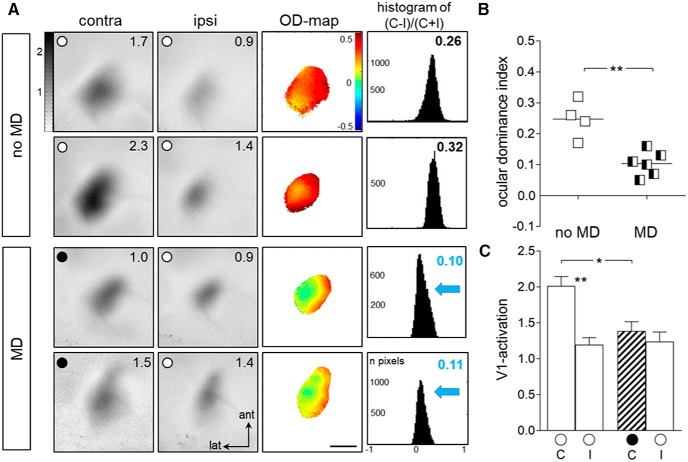
**. Transgenerational transmission of enhanced OD plasticity from EE mice to their non-enriched SC-raised offspring. *A***, Optically recorded activity maps of the contralateral (contra) and ipsilateral (ipsi) eye in the binocular part of V1 of SC-raised offspring of EE-parents without and with 7 d of MD. Data display and quantification as in Figure 1. V1 activity maps from two individual animals of each condition (no MD/MD) are illustrated. In mice without MD (no MD, top 2 rows), the activity patch evoked by visual stimulation of the contralateral eye is darker than the one of the ipsilateral eye, warm colors prevail in the two-dimensional OD maps and ODI values are positive. MD (bottom 2 rows) resulted in an OD shift toward the open eye so that both eyes activated V1 more similarly strong, colder colors appeared in the OD maps, and the ODI values were lower, i.e., the ODI histograms shifted to the left (blue arrows). ***B***, ***C***, Quantification of visual cortical activation before and after MD. ODI (***B***) and V1 activation (***C***) are illustrated. *p < 0.05, **p < 0.01.

Next, we visualized OD plasticity in V1 of the non-enriched offspring of EE-parents, i.e., in mice that were born and raised in SCs until at least P119, and thus never experienced EE-conditions on their own (see Materials and Methods for a detailed explanation of mating conditions). To trigger plasticity, half of the mice were subjected to 7 d of MD, whereas control mice from the same litter did not receive MD. As expected, V1 of mice without MD was dominated by the contralateral eye. In contrast, V1 of MD-mice was more equally activated by both eyes, and thus showed an OD shift (Fig. 2): in no-MD mice, the two-dimensional OD map showed warm colors, indicating contralateral dominance, whereas in MD-mice, colder colors prevailed the OD map and the OD histogram was shifted to the left (Fig. 2*A*).

Quantitative analysis of the ODI revealed that the adult non-EE offspring of EE-parents displayed an OD shift in V1 after MD implying that the plasticity promoting effect of EE can be transferred to the next generation (Fig. 2*B*). Specifically, non-EE mice from EE-parents had a mean ODI of 0.26 ± 0.02 (*n* = 4, P119–P209), reduced to 0.10 ± 0.02 after 7 d of MD (*n* = 6, P118–P261; *p* = 0.0019, ANOVA). As expected, the binocular part of V1 in mice without MD was activated more strongly after contralateral than after ipsilateral eye stimulation (*p* = 0.0062, ANOVA), whereas after MD, the V1 activation was similar after each eye stimulation (*p* > 0.999, ANOVA). The average ODI of monocularly deprived mice of the EE-parents group was significantly lower than that of the MD mice of the SC group (Fig. 1; 0.27 ± 0.04, *n* = 4, *p* = 0.0019, *t* test). Notably, the observed OD shift was mediated by a reduction of deprived (contralateral) eye responses in V1: V1 activation after contralateral eye stimulation was 2.02 ± 0.13 in mice without MD, and decreased to 1.39 ± 0.13 after MD (*p* = 0.0237, ANOVA). In contrast, open (ipsilateral) eye responses remained unchanged between no-MD and MD mice (no-MD/MD: 1.20 ± 0.10/1.24 ± 0.13, *p* = 0.997, ANOVA; Fig. 2*C*).

### OD plasticity was preserved in the offspring of EE-mothers, but not of EE-fathers

Next, we were interested in investigating whether both parents contribute equally to the observed transgenerational plasticity. To do this, we arranged matings between EE-males and SC-females, and between SC-males and EE-females. Offspring of both pairings were born and raised in SCs until at least P127. Thereafter, OD plasticity was assessed by intrinsic signal optical imaging as before.

**Figure 3:**
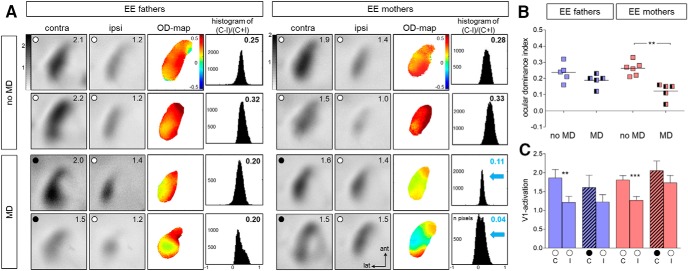
**. Adult non-enriched offspring of EE-mothers display OD plasticity, but not non-enriched offspring of EE-fathers. *A***, Optically recorded activity maps elicited by visual stimulation of the contralateral (contra) or ipsilateral (ipsi) eye in the binocular part of V1 of SC-raised offspring of EE-fathers (left column) or EE-mothers (right column) without (no MD), and with 7 d of MD. Data display and quantification as in Figure 1. V1 activity maps from two individual animals of each condition (no MD/MD) are illustrated. In mice without MD, the activity patches evoked by visual stimulation of the contralateral eye are darker than those of the ipsilateral eye, warm colors (red) prevail in the two-dimensional OD maps and ODI values are positive. After MD in offspring of EE-fathers, V1 remained dominated by the deprived (contralateral) eye in, whereas offspring of EE-mothers displayed OD plasticity, i.e., both eyes activated V1 more equally strong and the ODI histogram shifted to the left (blue arrows). ***B***, ***C***, Quantification of visual cortical activation before and after MD: both ODI (***B***) and V1 activation (***C***) are illustrated. Statistical significance was calculated using ANOVA and *p* values were corrected for multiple comparisons. **p < 0.01, ***p < 0.001.

As expected, visual stimulation showed that the binocular part of V1 of non-deprived adult offspring of both pairings (EE-mother/SC-father and SC-mother/EE-father) was dominated by the contralateral eye (Fig. 3): V1 activation was always stronger after contralateral eye stimulation compared with ipsilateral eye stimulation, the average ODI was positive, and warm colors prevailed in the two-dimensional OD maps. After MD of the contralateral eye in non-enriched mice born to EE-father/SC-mother pairings, the ODI did not change: the ODI remained positive, and warm colors continued to dominate the two-dimensional OD map. In contrast, in non-enriched mice born to EE-mother/SC-father pairings, a 7 d MD induced OD shifts: V1 was activated more equally strongly by visual stimulation of either eye, the ODI values were closer to zero, colder colors appeared in the OD map, and the OD histogram shifted to the left (Fig. 3*A*). Specifically, non-enriched offspring of EE-fathers displayed an ODI of 0.24 ± 0.03 (*n* = 5, P127–P188) which was not significantly changed after MD (0.19 ± 0.02; *n* = 5, P132–P194; *p* = 0.81 ANOVA). In contrast, non-enriched offspring of EE-mothers displayed OD plasticity: the ODI was 0.26 ± 0.02 (*n* = 6, P142–P188), and reduced to 0.12 ± 0.02 after MD (*n* = 5, P166–P177; *p* = 0.0011, ANOVA; Fig. 3*B*). Furthermore, V1 responses after stimulation of each eye were quantified (Fig. 2*C*). In non-enriched offspring of EE-father/SC-mother pairings, V1 activation after both contralateral (contra) and ipsilateral (ipsi) eye stimulation did not change after MD (contra, without/with MD: 1.83 ± 0.18/1.61 ± 0.32; p = 0.985, ANOVA; ipsi, without/with MD: 1.24 ± 0.13/1.23 ± 0.19, p = 0.969, ANOVA). In non-enriched offspring of EE-mother/SC-father pairs, V1 activation after contralateral eye stimulation was 1.81 ± 0.11 without MD and 2.05 ± 0.25 with MD (*p* = 0.985, ANOVA), whereas V1 activation after ipsilateral eye stimulation was increased from 1.27 ± 0.10 without MD to 1.73 ± 0.19 after MD. Although this increase of open eye responses in V1 was not significant (*p* = 0.052, ANOVA), it is worth noting that the OD shift of the non-enriched mice born to EE-mother/SC-father pairs was clearly not mediated by a reduction of deprived eye responses in V1, as observed for the non-enriched pups of EE-parents (compare Figs. 2*C*, 3*C*, rightmost histogram) and for adult EE-mice (Fig. 1). Differences between no-MD mice of both groups were not significant (*p* > 0.05, ANOVA).

Together, our data demonstrate that (1) in a primary sensory cortex, the plasticity promoting effect of EE can be transmitted to the next non-enriched generation, and (2) the life experiences of the mother have a greater impact on the continued V1 plasticity of the non-enriched SC offspring.

### Basic Visual abilities and enhanced optomotor reflex after md were similar in all experimental groups

In addition to the assessment of OD plasticity, spatial vision of all experimental animals was measured daily using the virtual reality optomotor setup (Prusky et al., 2004). First, baseline spatial frequency and contrast sensitivity thresholds of the optomotor reflex were determined in all mice of the three major experimental groups (non-enriched SC-offspring of EE-parents or EE-father/SC-mother or EE-mother/SC-father pairs).

Spatial frequency thresholds of adult non-EE mice born to EE-parents were 0.38 ± 0.001 cyc/deg (*n* = 11), values of mice born to EE-fathers were 0.38 ± 0.001 cyc/deg (*n* = 11), and the offspring of EE-mothers had a threshold of 0.39 ± 0.001 cyc/deg (*n* = 9); i.e., values did not differ significantly between the groups (*p* = 0.392, ANOVA).

Baseline contrast sensitivity thresholds of the optomotor reflex were also determined for the three major mouse groups at six different spatial frequencies (0.031, 0.064, 0.092, 0.103, 1.192, and 0.272 cyc/deg): contrast sensitivity values were again similar for all the groups (*p* > 0.05 for every spatial frequency and comparison, ANOVA).

**Figure 4:**
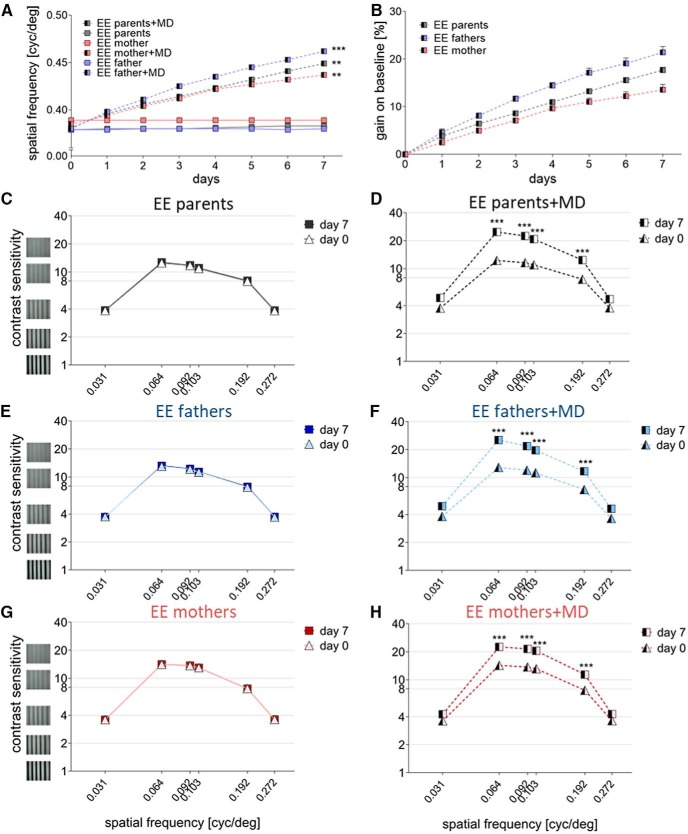
**. Experience-enabled improvements of the optomotor reflex where not different in non-enriched offspring of EE-parents, EE-fathers, and EE-mothers. *A***, ***B***, The spatial frequency threshold in cyc/deg (A) and gain on baseline (in %; ***B***) of the improvements of the optomotor reflex plotted as a function of days, over 7 d of MD or noMD. ***C***–***H***, Contrast sensitivity thresholds of the optomotor reflex on days 0 and 7 after MD (***D***, ***F***, ***H***) or for the no MD-period (***C***, ***E***, ***G***) at six different spatial frequencies. Note that both the spatial frequency and the contrast thresholds increased after MD in all three groups of non-enriched offspring. Statistical significance was calculated using ANOVA and *p* values were corrected for multiple comparisons. **p < 0.01, ***p < 0.001.

Afterward, MD was performed in some of the animals of each experimental group and the spatial frequency and contrast sensitivity thresholds of the optomotor reflex were tested daily in the following 7 d. As expected from previous research (Prusky et al., 2006), both spatial frequency and contrast thresholds of the open eye increased in all groups after MD. These data functionally confirmed that the MD eyes remained closed throughout the 7 d MD period, and served as an additional check for effective eye closure. In SC-mice born to EE-parents, the highest spatial frequency eliciting an optomotor response on Day 7 after MD was 0.45 ± 0.003 cyc/deg (*n* = 7), and thus significantly higher (*p* = 0.0011, ANOVA) than the values before MD (0.39 ± 0.001 cyc/deg, *n* = 5). In SC-mice born to EE-fathers, the spatial frequency threshold increased to 0.46 ± 0.002 cyc/deg on Day 7 after MD (*n* = 5), significantly higher than the baseline value before MD (0.38 ± 0.001 cyc/deg, *n* = 6; *p* = 0.0007, ANOVA). Likewise, in SC-mice born to EE-mothers, the threshold increased to 0.44 ± 0.003 cyc/deg after MD (*n* = 5), whereas SC-offspring of EE-mothers without MD had a value of 0.39 ± 0.002 cyc/deg on Day 7 (*n* = 5, *p* = 0.0043, ANOVA; Fig. 4*A*). Spatial frequency thresholds of all MD-groups increased significantly from Day 0 to Day 7 (*p* < 0.001 for EE-parents and EE-fathers, *p* < 0.01 for EE-mothers, ANOVA). In more detail, thresholds increased by 18%, for SC-offspring of EE-parents, by 21% for SC-offspring of EE-fathers and by 14% for SC-offspring of EE-mothers (Fig. 4*B*). Without MD, spatial frequency thresholds remained constant over time (*p* > 0.05, ANOVA).

Similarly, contrast sensitivity thresholds of all mice without MD remained constant over the 7 d of measurement (*p* > 0.05 for every frequency in each group, ANOVA; Fig. 4*C*,*E*,*G*), whereas contrast sensitivity thresholds of the optomotor reflex of the open eye increased significantly after MD compared with Day 0 in all three groups of SC-offspring for 4 of 6 of the tested spatial frequencies. In detail, statistical analyses revealed the following *p* values (ANOVA) comparing contrast thresholds on Day 0 before MD with Day 7 after MD for 0.031/0.064/0.092/0.103/0.192 and 0.272 cyc/deg): SC-offspring of EE-parents: *p* > 0.05/*p* < 0.001/*p* < 0.001/*p* < 0.001/*p* < 0.01/*p* > 0.01 (Fig. 4*D*), SC-offspring of EE-fathers *p* > 0.05/*p* < 0.001/*p* < 0.001/*p* < 0.001/*p* < 0.01/*p* > 0.05 (Fig. 4*F*), and SC-offspring of EE-mothers: *p* > 0.05/*p* < 0.001/*p* < 0.001/*p* < 0.001/*p* < 0.01/*p* > 0.05 (Fig. 4*G*). The values on Day 7 were not different between the three groups (*p* > 0.05 for every frequency and group comparison, ANOVA; Table 3).

**Table 3:** Contrast sensitivity values of mice born in SC from EE-parents, EE-father, or EE-mother on Day 7 of MD/no MD period

**Spatial frequency** **(cyc/deg)**	**EE-parents** **(*n* = 4)**	**EE-parents** **MD** **(*n* = 7)**	**EE-fathers** **(*n* = 6)**	**EE-fathers** **MD** **(*n* = 5)**	**EE-mothers** **(*n* = 5)**	**EE-mothers** **MD** **(*n* = 4)**
0.031	3.8 ± 0.35	4.8 ± 0.18	3.7 ± 0.08	4.9 ± 0.12	3.6 ± 0.04	4.3 ± 0.06
0.064	12.9 ± 3.26	24.9 ± 1.12	13.3 ± 0.21	25.9 ± 0.51	14.1 ± 0.37	22.6 ± 0.27
0.092	11.9 ± 2.64	22.5 ± 0.89	12.3 ± 0.20	22.0 ± 0.44	13.7 ± 0.38	21.5 ± 0.34
0.103	11.4 ± 2.44	20.8 ± 0.69	11.4 ± 0.09	20.2 ± 0.31	13.0 ± 0.31	20.5 ± 0.38
0.192	8.2 ± 1.31	12.4 ± 0.26	7.7 ± 0.12	11.7 ± 0.28	7.7 ± 0.15	11.4 ± 0.25
0.272	3.8 ± 0.27	4.7 ± 0.11	3.7 ± 0.11	4.7 ± 0.09	3.6 ± 0.03	4.3 ± 0.03

For the six different spatial frequencies tested the average contrast sensitivity for each group is listed as mean ± SEM.

In summary, after MD, mice from all experimental groups showed similar increases both in spatial frequency and contrast sensitivity thresholds. Thus, in contrast to cortical OD plasticity, enhanced optomotor responses after MD do not seem to be modified by the rearing conditions of the animals’ parents.

## Discussion

There is growing evidence that the effects of an enriched environment can be long-lasting, and even include transmission of acquired features to the next generation (Arai and Feig, 2011). Surprisingly, we observed such a transgenerational transmission of increased neuronal plasticity in a primary sensory cortical area. After raising mice in EE cages, OD plasticity in V1 was not only preserved into old age, in contrast to SC-raised mice, but most interestingly also in their adult non-enriched offspring; i.e., in mice that never experienced enrichment directly. The OD plasticity visualized in V1 of adult non-enriched offspring of EE-parents was mechanistically similar to the OD plasticity of adult EE-mice. In both experimental groups, the OD shifts were primarily mediated by a reduction of V1 activation after visual stimulation of the (previously) deprived eye. Testing non-enriched SC-offspring of enriched mothers and non-enriched fathers versus enriched fathers and non-enriched mothers revealed that enriched mothers seem to have a greater contribution to the transgenerational effect. Whereas the adult offspring of enriched mothers and non-enriched fathers displayed OD plasticity after monocular deprivation, non-enriched offspring of enriched fathers and non-enriched mothers did not significantly change V1 activation despite the 7 d MD period. Notably, however, there is some mechanistic difference between the OD plasticity observed in EE-mice or non-enriched offspring of EE-parents and non-enriched offspring of EE-mothers/nonEE-fathers. Although the OD shift of the former two experimental groups (EE-mice and SC-offspring of EE-parents) is primarily mediated by a reduction of deprived eye responses in V1 after 7 d of MD, this was clearly not the case in the SC-offspring of EE-mothers: optical imaging rather revealed a small but insignificant (*p* = 0.052) increase in open eye V1 activation. Because a reduction of deprived eye responses in V1 after 7 d of MD is characteristic for OD plasticity in adult enriched mice (Greifzu et al., 2014), a similar kind of OD plasticity is transmitted to the next generation only if both parents are enriched. Thus, the life experiences of the father must also contribute to the offspring’s cortical plasticity. Future *in vivo* spike recordings should reveal the mechanisms underlying these differences in OD plasticity, depending on whether only the mother or the father or both parents were raised in an enriched environment.

Together our data suggest that the supporting effect of EE on preserving OD plasticity into late adulthood can be passed on to the next generation, even if the offspring of EE-mice have never experienced EE-conditions on their own. Furthermore, the life experiences of the mother seem to have a greater impact on the continued OD plasticity of the offspring. We suggest that the increased OD plasticity observed in the non-enriched offspring of EE-parents may arise because of inherited epigenetic changes and modified behavior, e.g., mother–pup interactions.

Epigenetic changes have been shown to affect the neural development of the offspring in previous studies. It was reported that pregnant rats exposed to enriched environment showed enhanced ability to find their way in a maze as well as the ability of their offspring to do the same (Kiyono et al., 1985). In another study, female mice exposed to EE showed enhanced learning ability that was transmitted to their offspring even if the exposure to EE was before pregnancy (Dell and Rose, 1987). Their observations were similar when non-EE foster mothers raised the offspring of EE-mothers, suggesting that the effect of EE was transmitted to the offspring before birth, presumably *in utero*. In support of these data, Arai et al. (2009) showed that 4-week-old offspring of EE-mice displayed enhanced synaptic plasticity in the hippocampus, as their EE-raised parents. Interestingly, only the mother’s contribution to the transgenerational transmission of the EE-effect was significant. The enhanced hippocampal plasticity was also observed when the offspring of EE-mothers were raised from birth by a non-EE foster mother, suggesting that the effect of EE was transmitted to the offspring before birth (Arai et al., 2009). It is possible that *in utero* exposure to EE had a plasticity promoting effect also in our study; however, because the OD shifts in offspring of EE-parents and EE-mothers were mechanistically different, *in utero* exposure is likely only a contributing factor and cannot be accounted for all the observed effects. Future experiments with non-EE foster mothers should clarify whether the prolonged OD plasticity is because of specific behavior of an enriched mother or due to *in utero* epigenetic modifications. In this context, it would also be interesting to investigate the expression profile and the chromatin remodeling of plasticity-related genes after exposure to EE and to test whether any modifications can be transmitted to the next generation.

It is well known that rodent mothers play a big role for both prenatal and postnatal development of their offspring (Liu et al., 2000). Mothers provide nutritional and behavioral support by, e.g., licking and grooming, which may have long lasting beneficial effects for brain plasticity (Champagne et al., 2003). Furthermore, mothers raised in EE provide their pups with higher levels of maternal care compare to SC-raised mothers, which may also explain the long-lasting plasticity observed in their offspring (Baroncelli et al., 2010). During the first 2 weeks of life, rodents stay in the nest, totally depending on the mother, which is the most important source of sensory experience (Ronca et al., 1993). EE-pups receive higher levels of maternal care, continuous physical contact, and higher levels of licking compared with SC-reared pups (Sale et al., 2004). It has been proposed that higher maternal care in EE-pups affects their visual system development (Cancedda et al., 2004), possibly because of increased BDNF levels in the offspring caused by variations in maternal care (Liu et al., 2000). Mimicking part of maternal behavior during the first 10 d of a rat pup’s life by using tactile stimulation (massage), a procedure previously shown to compensate for the negative effects of maternal deprivation (Schanberg and Field, 1987), was able to reproduce the EE-dependent acceleration of visual development in rat pups born in SCs (Guzzetta et al., 2009). The effect of tactile stimulation was attributed to increased IGF-I levels in P18 pups, as blocking IGF-I abolished the EE-induced acceleration of visual system development (Guzzetta et al., 2009). IGF-I signaling could therefore be a contributing factor to the continued OD plasticity of EE-mice and their non-enriched offspring observed in the present study.

Furthermore, the level of maternal care can also influence the response to stress in adulthood (Meaney, 2001). High levels of maternal care results in elevated levels of hippocampal serotonin in the pups, leading to increased expression of the transcription factor NGFI-A binding to the glucocorticoid (GR) promoter. Higher levels of NGFI-A result in increased expression of the GR-receptor through posttranslational modifications like DNA hypomethylation and histone acetylation which is correlated with reduced stress levels. These epigenetic modifications are maintained into adulthood and are heritable, marking the level of maternal care on gene expression patterns across generations (Weaver et al., 2004).

Although the mother’s role in the fitness of offspring has been long recognized, there is also increasing literature on epigenetic transmission of neurobehavioral phenotypes through the paternal line (for review, see Yuan et al., 2016). The fact that the OD shift in the offspring of EE-mothers and SC-fathers was not the same as in the offspring of EE-parents (or in adult EE-mice) already indicates that the transgenerational transmission of OD plasticity cannot solely be explained by any epigenetic/behavioral changes of mothers, but that epigenetic transmission through the paternal and/or maternal line must also be involved. Therefore, epigenetic influences and modified maternal care likely work in concert to support the transgenerational transmission of the increased OD plasticity.

In addition to imaging cortical OD plasticity, we assessed the basic visual capabilities of mice by measuring the spatial frequency and contrast thresholds in the optomotor setup before and during monocular deprivation. Neither of the measured parameters differed between the three major experimental groups (EE-parents, EE-mothers/SC-fathers, EE-fathers/SC-mothers) before or during the MD, and values were also similar to previously published data from similarly aged SC- and EE-mice (Prusky et al., 2006; Greifzu et al., 2014). Although it was shown that EE has a positive effect on early visual system development (Cancedda et al., 2004), we did not observe any enhancement in the optokinetic reflexes in any of the examined groups.

The observation that MD-induced optomotor enhancements do not depend on the raising conditions of the mice or their parents suggests that life experiences affect the underlying neuronal circuits differently compared with OD plasticity. It is known that the optomotor response is mediated by the brainstem accessory optic system, whereas cortical circuits are necessary for the MD-induced increases in optomotor thresholds (Prusky et al., 2006). Specifically, it was recently shown that cortico-fugal projections from V1 to the accessory optic system, in particular to the optic tract and dorsal-terminal nuclei (NOT-DTN), underlie learning-induced optokinetic reflex potentiation (Liu et al., 2016). Thus, experience-induced optomotor enhancements and OD plasticity in V1 depend on different nerve cell circuits. The results of these two “plasticity” paradigms can therefore be independent of each other and must not follow similar rules. In fact, it was shown previously that results can dissociate: whereas ibuprofen treatment could rescue the deprivation-induced enhancement of optomotor thresholds in mice with a cortical stroke in the primary somatosensory cortex, it did not rescue OD plasticity in the same animals (Greifzu et al., 2011).

Genetic programs, epigenetic information, and experience work in concert to optimize the development and behavior of an organism for survival in a particular environment. There is now ample evidence that, in addition to the genes transferred to the next generations by germ lines, experiences of the parents can markedly influence both structure and function of the nervous system and behavior of subsequent generations through inherited epigenetic modifications (Arai and Feig, 2011). Our work provides additional support to this idea by showing that even in a primary sensory cortical area, a plasticity-promoting effect of an enriched environment, or less-deprived rearing, can be transferred to the next generation. Most likely based on the important role of the mothers in prenatal and early postnatal development of the offspring, mothers appear to have a slightly more important role than fathers in this transgenerational transmission. Importantly, our data further indicate that the outcome of any brain plasticity experiment might not only depend on the life experiences of the particular experimental animal but also can be influenced by the life experiences of the parents of this experimental animal, which therefore should be carefully documented. Further investigations are necessary to elucidate the exact mechanisms underlying this transgenerational rescue of brain plasticity and the differences in the magnitude and mechanisms of the experience-dependent V1 activation changes.

## Synthesis

Reviewing Editor: Tatyana Sharpee, The Salk Institute for Biological Studies Decisions are customarily a result of the Reviewing Editor and the peer reviewers coming together and discussing their recommendations until a consensus is reached. When revisions are invited, a fact-based synthesis statement explaining their decision and outlining what is needed to prepare a revision will be listed below. The following reviewer(s) agreed to reveal their identity: Jose Maya-Vetencourt, Tristan Chaplin.

This is a very interesting and ambitious study that to examine if neural plasticity can be inherited. However, a number of experiments are still needed to support the conclusions unequivocally. Also, it seems that one should be more cautious in claiming the transgenerational transmission if the results can be explained by increased maternal care from mothers raised in enriched environments or reduced stress in utero.

Reviewer 1:

In this work, the authors investigate the transgenerational transmission of plasticity by using the ocular dominance (OD) plasticity paradigm. It has been established that mice raised in an enriched environment (EE) display enhanced OD plasticity in adulthood. In the present work, the adult offspring of EE mice's OD plasticity was assessed following 7 days of monocular deprivation (MD) with intrinsic signal optical imaging of V1 and with a behavioral optomotor-reflex (oMR) test. The authors indicate that the offspring of EE mothers, but not fathers display lifelong OD plasticity. The experimental study is both simple and elegant, but the conclusions are not supported by the experimental data. A high number of fundamental points need to be addressed. The lack of appropriate controls (&gt;P200, see details below) makes this manuscript not suitable for publication in its present form. The subject, however, is interesting. I provide a list of suggestions in the hope to improve the quality of the manuscript.

1. One of the main's reviewer concerns is the age of the animals used in this work (P120). The authors report that OD plasticity is preserved in EE animals as much as in the non-EE off-spring (mice born to enriched parents but raised under standard conditions until P120). This is clearly a mistake as this type of plasticity (7 days of monocular deprivation) is still present in P100 animals (PLoS ONE 2008, 3(9): 33120). Only in &gt;P200 mice, 7 days of monocular deprivation is no longer able to promote functional modifications of neural circuitries in the visual cortex. The authors are advised to do their experiments, at least, in a small cohort of older animals (&gt;P200) per group. Otherwise, the contribution of residual plasticity to the experimental findings cannot be ruled out.

2. The authors claim that no OD plasticity is observed after 7 days of MD in P120 mice. This is clearly incorrect and suggests that the imaging technique used in this study is very likely not appropriate to assess this type of plasticity. The authors are strongly advised to analyze OD plasticity, in the same small cohort of animals suggested before, by means of in vivo electrophysiology.

3. 3 different sets of offspring were tested: EE parents, EE fathers and EE mothers. Although the plasticity of adults raised in standard (SC) cages has been extensively characterized in the literature, it is an essential control which was not performed in this study. Age-matched SC animals need to be tested in the same manner as the other sets and compared to them.

4. The main finding of the study, which is that EE mothers transfer their plastic abilities to their offspring originates from the intrinsic imaging data in figure 2B. The results from the oMR behavioral test do not provide any additional information to the script. The authors infer this test to be a valid model for testing OD from Prusky and colleagues' work in 2006, which reports an enhancement of visual acuity as a result of monocular deprivation. However, they do not apply the same rigor as Prusky and colleagues' study and only test the non-deprived eye.

5. In the discussion and introduction, the authors infer that behavioral influence of the EE mother affects the plasticity of their offspring. However, this is not based on hard evidence. To make such claims, the authors need to rule out such influences as that of genetics and epigenetics (see Neural Plasticity 2013; 2013: 605079; Journal Experimental Neuroscience 2013; 7: 75-83). This also highlights the lack of histological, molecular and biochemical data in this study.

Reviewer 2:

This study investigates ocular dominance (OD) plasticity that arises during monocular deprivation (MD). MD plasticity is not normally observed in adult mice raised in standard cage (SC) environments, only adult mice raised in enriched environments (EE). Surprisingly, the authors find that MD plasticity can be observed in adult SC mice that are the offspring of EE mice. Subsequent experiments find that the mother is primarily responsible for this result. Finally, the authors examine the visual abilities of these mice, and observe changes in spatial frequency thresholds and contrast sensitivities in MD mice with EE parents.

The study appears to have a good design based on large amounts of previous work, and utilizes a well established method and phenomena (MD plasticity). The authors undertook an ambitious and novel study in a new but controversial area of research (non-Mendelian transgenerational inheritance). The work appears to be of high quality and has carefully considered many of the methodological issues, and the behavioural study adds important insights.

However, I have major concerns that as the data are currently presented, the small sample size makes it difficult to reject the strong prior that plasticity effects are not “transmitted”, and that the paper relies on statistical tests of these very small samples and metrics. I think the study also should have had control groups of SC mice *without* EE parents, since the EE/SC status of the parent is the variable of interest. These experiments may have been done in other papers, but it does seem that such data would have been useful here.

Furthermore, there appear to some inconsistencies between the findings which raise doubts:

1) In Figure 1, contralateral activation decreases in MD, but this is not replicated in Figure 2. Of course, this does not need to be the case, but the author's themselves make a claim that their data shows that the effect of EE is transmitted by the mothers (see comment in the Introduction), when it does not appear be the same process.

2) The changes in visual abilities do not seem to depend on the parents, which appears to contradict Figure 2, and the claim about the role of the mothers.

General comments:

Apart from obvious option of collecting more data, I would suggest that more raw data of individual cases could be shown. I presume the maps are averaged across mice (see comment in Methods) and the histograms are based on these averaged maps. But I think that since there are not that many mice, and only 3 figures were used in this manuscript, there is ample opportunity to show more raw data to make a more convincing argument. For example, I think the authors could show the OD maps and histograms of at least some of the individual mice to give a better impression of the variability between animals and across V1 (since Figure 1A seems to show a mediolateral gradient which is unexplained). Error bars for the mean OD values (Figure 1B, 2B) could be obtained with bootstrapping. Furthermore, I think figure 1C/2C are quite important because it seems to show how the plasticity manifests, but it is unclear to me how variable this is between animals and across V1, and how well this matches previous studies.

Finally, throughout the paper there are various references to “transmission” of acquired features to the next generation, which seems to allude to an unknown epigenetic mechanism. But as the authors themselves point out and discuss in depth, EE mice provide higher levels of maternal care. Or for example, maternal stress could affect the development in utero, which would result in less radical interpretations of these data. I think the authors should justify the use of the “transmission” and clearly define it, since it seems that these results could simply be an extension of EE effects, rather than any feature being transmitted.

Specific comments:

Introduction:

Line 80 “Our data clearly show that i) in a primary sensory cortex, the plasticity promoting effect of growing up in a stimulating (or less deprived) environment not only affects the mice experiencing this enrichment, but also their future offspring”

- This seems misleading. As I see it, the paper does not show that EE affects plasticity, that was a previous study. This paper only shows the effects in the offspring of EE mice.

Line 83 “ii) an enriched mother seems to be responsible for the transmission of the effect.”

This seems too strong - perhaps “responsible” should be changed to “necessary”, since the effect observed in Figure 1 is not the same as in Figure 2.

Methods:

- It is unclear to me how the maps were calculated - they appear to be averages but I don't see how they were created (e.g. how were they aligned? How were they identified? How many pixels are there in each case and together? Were they thresholded? Why are they different shapes?).

- The methods should state the number of animals in each group including any that were discarded and reasons for doing so, since it is unclear why some groups have different numbers of animals and how the sample sizes were chosen.

Results:

Figure 1:

- It took me a while to understand Figure 1, mostly because I was expecting controls showing the effect of MD on SC and EE mice first, so I would know what to expect for the EE offspring data. I would suggest a cartoon hypothesis figure, either as part of this or preceding it, that gives some indication of what is expected, e.g. a normal EE OD map is all red, an MD EE OD map is more neutral, OD doesn't occur in SC, etc., or something to graphically outline the hypothesis.

- Although it was later stated in the discussion, I was not aware that reduction in contralateral activation is typical of EE raised MD mice, this should be stated earlier or included as part of the hypothesis clarification comment above.

- Figure 1 should clarify that “contra” and “ipsi” are referring to the eyes.

- I would prefer it if the number in the top right of the maps is labelled, at least in the first instance.

- I would prefer it if the circle in the top left is labelled, at least in the first instance,

- It seems “V1 activation” needs units, or a description in the methods of what it is.

- The colour scale could be improved/compressed - the blue values don't seem to be used.

Figure 2

- See comments about raw data, as well as those from Figure 1.

Figure 3:

- Is day 7 data missing from the left column? Or are they really identical to day 0? Perhaps this should be noted or better visualized.

- What is the inset brain showing? The colours don't seem to match anything?

- This section of the text should have a summary statement to say what overall findings of this figure are, especially in the context of MD plasticity.

Discussion:

- The discussion (and abstract) does not seem to cover the results from Figure 3, and how they relate to the imaging results, which cannot be ignored since they appear to be inconsistent.

- Line 349: “There is now ample evidence that, in addition to the genes transferred to the next generations by germ lines, experiences of the parents can markedly influence both structure and function of the nervous system and behaviour of subsequent generations through inherited epigenetic modifications (Arai and Feig, 2011). Our work provides additional support to this idea by showing that”

This seems to make an implicit link that the observations in this study are epigenetic, when it has already been outlined that factors such as maternal care could be important. Perhaps it would be useful to define epigenetics if the authors wish to use this term, as it is quite controversial.
